# Dr. George Roe Carter

**Published:** 1909-05-01

**Authors:** 


Dr. George Roe Carter, M.R.C.P.I., medical officer of health of the Penge District Council, died on April 26 at the age of 66 at his residence at Anerley, where for 30 years he had been engaged in an extensive practice. Dr. Carter spent the earlier years of his professional life at St. Lucia, West Indies, where he acted as colonial surgeon and medical officer of health to the island. He was educated throughout at Dublin, and was for a time house surgeon to the Meath Hospital. He was a Fellow of the Royal Society of Medicine (London) and a member of the council of the obstetrical section. He was also a Fellow of the Royal Institute of Public Health and an ex-member of the Councils of the British Gynæcological Society and the Irish Medical Graduates' Association.

